# Child and adolescent sleep disturbances and psychopathology in a mental health clinic sample

**DOI:** 10.3389/frsle.2024.1399454

**Published:** 2024-10-23

**Authors:** Aviva Blacher, Katarina N. A. McKenzie, Shannon L. Stewart, Graham J. Reid

**Affiliations:** ^1^Department of Psychology, The University of Western Ontario, London, ON, Canada; ^2^School and Applied Child Psychology, Faculty of Education, The University of Western Ontario, London, ON, Canada; ^3^Division of Children's Health and Therapeutics, Children's Health Research Institute, Lawson Health Research Institute, The University of Western Ontario, London, ON, Canada; ^4^Child and Adolescent Psychiatry, Schulich School of Medicine and Dentistry, The University of Western Ontario, London, ON, Canada; ^5^Department of Family Medicine, Schulich School of Medicine and Dentistry, The University of Western Ontario, London, ON, Canada; ^6^Department of Paediatrics, Schulich School of Medicine and Dentistry, The University of Western Ontario, London, ON, Canada

**Keywords:** sleep problems, internalizing, externalizing, interRAI, clinical sample

## Abstract

**Introduction:**

Children and adolescents treated in specialty mental health services are more likely to have sleep disturbances than those without mental health problems. Few studies have investigated the relationship between sleep and psychopathology in broad clinical samples of children. We examined the relationship between sleep disturbance and age on internalizing and externalizing psychopathology in a sample who sought treatment at children's mental health centers.

**Methods:**

Secondary data analyses were completed on a sample of children (*N* = 13,472; aged 4 to 18; 55% male) from 39 children's mental health agencies in Ontario, Canada, who completed a semi-structured assessment, the interRAI Children and Youth Mental Health (ChYMH). A split-half sample approach was utilized (S1 *n* = 6,773, S2 *n* = 6,699). Hierarchical regressions examined the effects of sleep disturbances (i.e., difficulty falling asleep, staying asleep, night waking, bedtime resistance, falling asleep during the day) on internalizing and externalizing symptoms, above and beyond established child- (i.e., age, sex, sensory sensitivity, pain) and family-level variables (family functioning, caregiver distress, parenting strengths). Age was tested as a moderator for sleep disturbances on both outcome variables.

**Results:**

Overall, 6.7% of children had clinically significant sleep disturbance scores (≥10 out of 16) on the interRAI ChYMH. In both samples, sleep disturbances predicted internalizing (S1 ΔR^2^ = 10%, S2 ΔR^2^ = 10%) and externalizing symptoms (S1 ΔR^2^ = 2%, S2 ΔR^2^ = 1%), above and beyond child and family variables. Age moderated the relationship between sleep disturbances and internalizing symptoms (S1 ß = 0.07; S2 ß = 0.07; ΔR^2^ = 0.004 in both samples), but not externalizing symptoms; sleep disturbance was more strongly related to internalizing symptoms amongst adolescents (ß = 0.98) than children (ß = 0.62).

**Discussion:**

The relationship between sleep and internalizing symptoms appears to change as children move through development. Further, sleep was a stronger predictor of internalizing problems in adolescents than children, suggesting an additional focus of clinician efforts in this age group. These findings strengthen the importance of routine assessment of sleep, as is done with the interRAI ChYMH.

## 1 Introduction

Sleep disturbances among children and adolescents are common, with prevalence rates of 10–25% in community samples, depending on the type of sleep problem and assessment methodology (e.g., Owens, 2007; Lewien et al., 2021; Ipsiroglu et al., 2002; Johnson et al., 2006). The most common sleep disturbances are problems getting to sleep (including bedtime resistance among younger children), and night waking. These issues can in turn lead to problems falling asleep during the day (excluding napping in those < 6 years of age). These types of sleep disturbances are associated with multiple short- and long-term impairments, including impaired cognitive and physical functioning and greater psychopathology (e.g., Alfano and Gamble, 2009; Chaput and Janssen, 2016; Paruthi et al., 2016; Van Dyk et al., 2016).

Sleep disturbances have been related to both internalizing (i.e., anxiety, depression) and externalizing (i.e., attention, behavior issues) psychopathology (Bagley and El-Sheikh, 2013). Sleep disturbance is common amongst adolescents suffering from depression (Reigstad et al., 2010), as well as children and adolescents suffering from different anxiety disorders (Alfano et al., 2007). A longitudinal study of children and adolescents treated for internalizing problems found a bidirectional relationship between sleep disturbance and symptoms of depression and anxiety (Bai et al., 2020). Similarly, in community (e.g., Quach et al., 2018; Liu et al., 2022)and clinical samples, sleep problems are common amongst individuals with behavior problems and ADHD (Corkum and Coulombe, 2013).

The relationship between sleep problems and childhood psychopathology is complex and bidirectional (e.g., Beebe, 2011; Van Dyk et al., 2016; Vazsonyi et al., 2022). Psychopathology may lead to, or exacerbate, sleep problems, while sleep disturbances may interfere with emotion regulation and lead to mental health difficulties; or both sleep and psychopathology may interact, impairing a child's functioning in multiple domains across an extended period of time (Alfano and Gamble, 2009; Gregory and Sadeh, 2012).

Few studies have investigated sleep disturbances in broad clinical samples of children and adolescents in mental health services, and most studies recruited cases coming from just one clinic. Some studies have focused on extreme groups, such as psychiatric inpatients (Johnsen et al., 2024; Boafo et al., 2021). Other studies have looked at individuals with specific disorders such as anxiety (Bai et al., 2020; Chase and Pincus, 2011) or depression (Orchard et al., 2017; Manglick et al., 2013). The few studies that examined clinical samples with a range of disorders often compared clinical cases to community controls, and then examined associations between sleep and psychopathology. An interesting study from Norway linked epidemiologic data with health administrative data and found higher rates of insomnia among adolescents who had involvement with child and adolescent mental health services compared to the rest of the population (Hysing et al., 2022). Reigstad et al. (2010) compared sleep in 129 adolescents (ages 13–17) receiving outpatient mental health services with adolescents in the community. Sleep disturbances (e.g., difficulty sleeping, feeling overtired) were experienced by 37% of community controls, compared to 80% of psychiatric outpatients. More sleep disturbances were associated with greater internalizing problems in both outpatients and the community. Further, in the clinical sample, sleep problems related to poorer family functioning (Reigstad et al., 2010). Simonds and Parraga (1984) investigated the frequency of sleep disturbances in 150 children and adolescents (ages 4–18) receiving outpatient mental health services compared to children in the general population. Among the clinical sample, restless sleep was most common in patients with anxiety or conduct disorders, or ADHD. Patients with ADHD experienced the greatest number of sleep difficulties. Ivanenko et al. (2004, 2006) had different findings amongst 174 children and adolescents (ages 5–18) receiving care at an outpatient mental health clinic, who were compared to a control group (ages 5–16). Children with mood and anxiety disorders alone or ADHD comorbid with other psychopathology had more night awakening than ADHD alone or other psychiatric diagnoses.

The literature to date clearly demonstrates a relationship between sleep disturbances and psychopathology in samples of children and/or adolescences receiving mental health care. As noted, key limitations to this literature include the tendency to study samples from only one clinic, samples limited to either children or adolescents but not both, and few studies of cases having a wide range of psychopathologies. Where both children and adolescents were included in the sample, rarely have differences by age been examined. A related point is that parents may not be as aware of their adolescent's sleep problems, compared to children. Including adolescents' report of their sleep issues addresses this. In addition, when examining the relationship between sleep and psychopathology rarely have correlates of sleep and mental health problems been controlled for. The current study addresses these limitations. Below, we briefly review the rationale for the correlates of sleep and psychopathology we controlled for.

### 1.1 Child age

Children need more sleep than adolescents or adults (Meltzer and Mindell, 2006). Further, age is related to the prevalence of sleep problems. A longitudinal study investigating sleep problems in a community sample found that overall sleep disturbances decreased from age 4 to mid-adolescence (Gregory and O'Connor, 2002). Age may also moderate the relationship between sleep and psychopathology, given that development is marked by structural and organizational changes in sleep-wake patterns (Feinberg and Campbell, 2010). Age-related changes have been observed in the relationship between sleep disturbances and internalizing disorders such as depression and anxiety (Benca et al., 1992; Johnson et al., 2000; Kelly and El-Sheikh, 2014). A meta-analysis by Lovato and Gradisar (2014) found the association between sleep disturbances and depression was stronger for adolescents than children. In contrast, the relationship between sleep disturbances and externalizing disorders does not seem to alter with age. A longitudinal study of children age 4 to mid-adolescence found that there was no significant age-related change in the correlation between sleep and aggression (age 4, *r* = 0.45; mid-adolescence, *r* = 0.40; average correlation across the 11-years, *r* = 0.38) or sleep and attention problems (age 4, *r* = 0.38; mid-adolescence *r* = 0.46; average correlation across the 11-years, *r* = 0.37) (Gregory and O'Connor, 2002). Given the paucity of research in this area we examined age as moderator for both internalizing and externalizing problems.

### 1.2 Risk factors for sleep problems

There is a vast literature on risk and protective factors in the development of psychopathology (e.g., Grizenko and Fisher, 1992; Pinto et al., 2014; Wille et al., 2008). Further, numerous factors have been shown to contribute to both sleep and psychopathology at the individual- and family-level (Bartel et al., 2015; Newton et al., 2020).

### 1.3 Individual-level factors

#### 1.3.1 Child sex

Child sex has also demonstrated important relationships to psychopathology and sleep disturbance. Females tend to have higher rates of internalizing problems than males, with marked differences occurring during adolescence (Georgiades et al., 2019; Crick and Zahn-Waxler, 2003). During childhood, boys are more likely than girls to have externalizing problems (Crick and Zahn-Waxler, 2003). With respect to sleep, girls tend to have longer sleep durations with less fragmented sleep than boys (Franco et al., 2020). A recent review found that sex did not to relate to sleep problems amongst children (Newton et al., 2020). However, during adolescence, sex differences begin to emerge and females are at risk for insomnia throughout adulthood (Zhang and Wing, 2006; Marver and McGlinchey, 2020). Thus, we examined sex as a correlate of sleep problems and the interaction of Sex X Age (in children and adolescents).

#### 1.3.2 Sensory processing

Sensory processing may impact sleep and psychopathology (Shochat et al., 2009; Reynolds et al., 2012). This includes sensation seeking (creating noise or movement like rocking back and forth that interferes with daily routine) and tactile sensitivity (reacting emotionally or sensitively to touch) (Tomchek and Dunn, 2007). In healthy children, tactile sensitivity has been related to greater sleep disturbances (e.g., bedtime resistance, night waking) and both tactile sensitivity and sensation seeking have been related to greater behavioral and emotional problems (e.g., impulsiveness, mood swings, frustration, and social difficulty) (Shochat et al., 2009). Atypical sensory behaviors, such as tactile sensitivity, are associated with lower sleep quality in both children with and without autism (Foitzik and Brown, 2018; Reynolds et al., 2012; Shochat et al., 2009).

#### 1.3.3 Pain

Higher levels of pain are associated with poorer sleep quality and depressive symptoms in both community samples and clinical samples of children with chronic pain (Siu et al., 2012). Shorter sleep duration and poorer subjective sleep quality have also been associated with greater pain the following day (Lewandowski et al., 2010).

### 1.4 Family-level factors

#### 1.4.1 Positive parenting

Positive parenting refers to behaviors that improve the relationship between parents and their children, such as communication, warmth, support, and supervision. Several studies have found that positive parenting is related to positive outcomes in children, including increased sleep duration (Sadeh et al., 2002; Philips et al., 2014). Parental warmth and emotional security have also been related to increased total sleep time among school-aged children (Adam et al., 2007; El-Sheikh et al., 2007a).

#### 1.4.2 Family stressors

Family dysfunction and parental psychological difficulties have been associated with sleep disturbances and psychopathology in children (Seifer et al., 1996; Lavigne et al., 1999; Reigstad et al., 2010; Gregory and O'Connor, 2002). Several studies using structural equation modeling have found higher family stressors lead to more sleep disturbances, in turn contributing to greater behavioral problems (Bates et al., 2002; El-Sheikh et al., 2007b; Goodnight et al., 2007; Reid et al., 2009).

### 1.5 Objectives and hypotheses

The current study aimed to examine: (1) the relationship between sleep disturbance on internalizing and externalizing psychopathology, above and beyond the impact of individual- and family-covariates in a clinical sample of children 4–18 years old; and (2) the interaction between age and sleep problems, and (3) the three-way interaction of age, sex and sleep disturbances on psychopathology.

Hypotheses: (1a) Individual- and family-level covariates were expected to predict higher internalizing (e.g., female sex, older age) and externalizing (i.e., male sex, younger age) symptoms. (1b) After controlling for individual- and family-covariates, higher levels of sleep problems were expected to be related to higher levels of both externalizing and internalizing psychopathology.

Hypotheses: (2a) It was expected that the relationship between sleep problems and internalizing would become stronger with increasing child age. (2b) For externalizing, we expected that the relationship with sleep problems would become weaker with increasing child age. (3) We expected the three-way interaction of age, sex and sleep disturbance would be stronger for girls than boys for internalizing symptoms and stronger for boys than girls on externalizing symptoms.

## 2 Methods

Secondary data analyses of administrative data from 2012 to 2018 children's mental health agencies in Ontario, Canada were completed for the present study. Publicly-funded specialized children's mental health services for up to 18 years of age in Ontario were delivered in community agencies (Duncan et al., 2018). Agencies provide a wide range of services. Access and services received are based on need; insurance coverage, and the ability to pay out of pocket. Having a diagnosis does not influence services.

The interRAI Child and Youth Mental Health is a semi-structured assessment instrument designed to assist with care planning, connection to services, and outcome tracking (Further information see Stewart et al., 2022). It consists of 400 items that assess areas of risk, child strengths and weaknesses, medical issues, and family dynamics. The interRAI ChYMH uses a combination of various sources such as self-report, parent-report, teacher-report, medical records, and clinical observation where appropriate. All clinicians administering the interRAI ChYMH undergo standardized training and must pass an online test. The interRAI ChYMH assessment is conducted by a trained clinician with the assistance of an interpretation guide that outlines question intent, definitions of key terms, time frame under consideration, and specific scoring systems. Informed consent was obtained from guardians of the children as part of the standard clinical care at each respective agency across Ontario. As part of an ongoing data sharing agreement, data from all agencies using the interRAI ChYMH are stored in an anonymized interRAI database intended for research purposes. No information is available on the percentage of clients at each agency who did not complete the interRAI ChYMH assessment.

**Figure 1:**
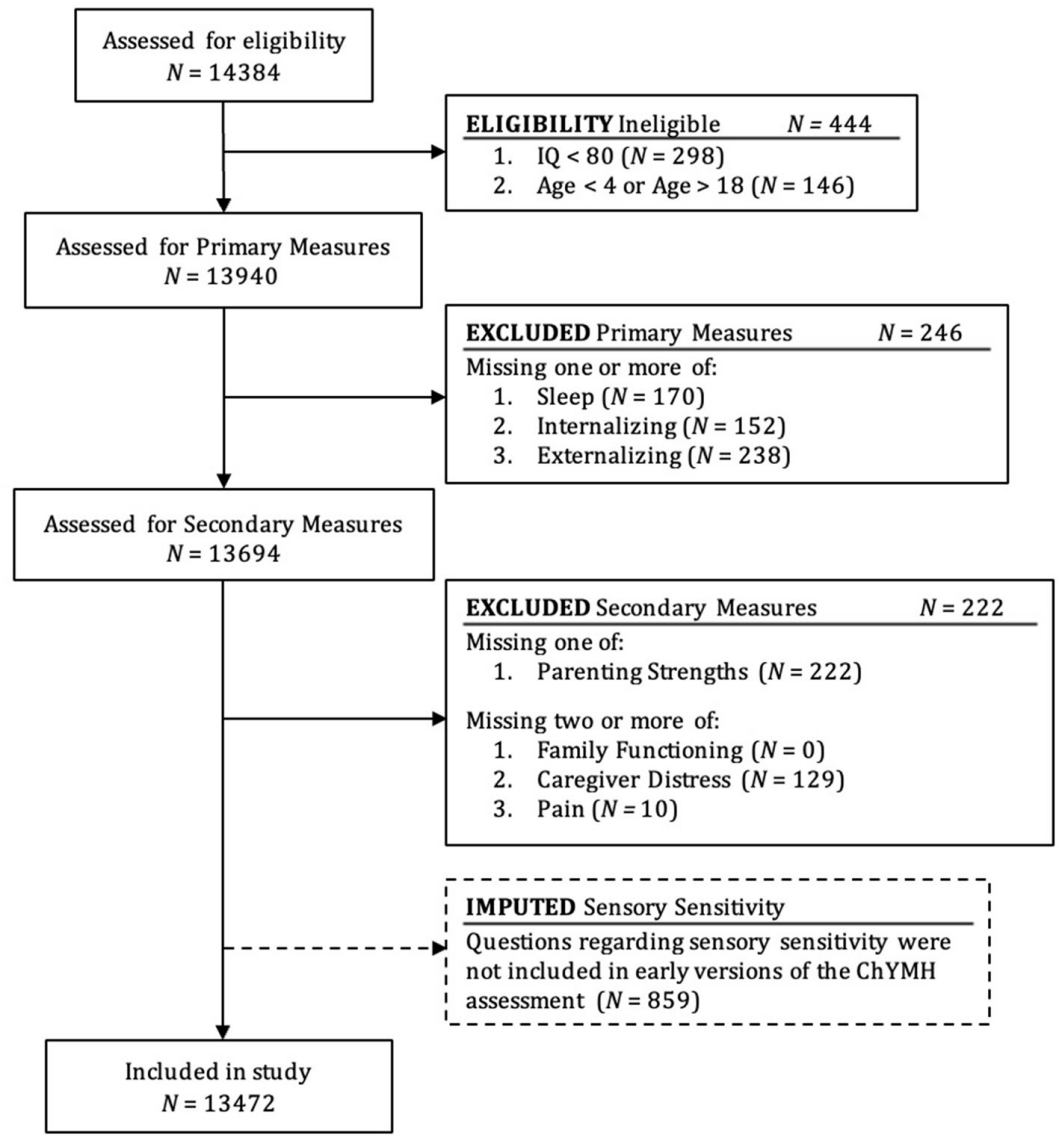
Flow chart showing selection of cases included in analyses.

A unique Case Record Number (CRN) is used to identify interRAI ChYMH records within an agency. For this study, only the initial interRAI ChYMH record was examined. A total of 14,384 children from 4 to18 years old and their families completed the interRAI ChYMH assessment as part of standard care in residential or outpatient settings across 39 mental health agencies from across the province of Ontario, Canada. Study criteria included selection based on clinical/demographic characteristics and availability of relevant measures (see Figure 1). In the current study, children were excluded if it was determined that they had an intellectual or learning disability (IQ < 79, *N* = 298). Children were also excluded if they fell outside of the intended age 4–18 year range of the interRAI ChYMH comprehensive assessment (*N* = 146). Composite measures of internalizing and externalizing were used, in line with current conceptualizations of psychopathology (Forbes et al., 2016; Lahey et al., 2017; Fusar-Poli et al., 2019).

### 2.1 Outcomes

#### 2.1.1 Internalizing scale

The Internalizing scale consists of 3 subscales: anxiety, anhedonia, and depression (Lau et al., 2019). Each item is scored from 0 to 4 based on presence over the last 3 days (*0* = *not present; 4* = *exhibited daily in last 3 days, 3 or more episodes or continuously*). An internalizing score was computed by summing items; scores range from 0 to 48 with greater scores indicating greater levels of internalizing symptoms. The internalizing scale has demonstrated strong content (i.e., expert panel) and criterion validity with internalizing scores from other measures (e.g., Child Behavior Checklist, *r* = 0.62). Internal consistency for the internalizing scale in this study was good: Cronbach's α = 0.85.

#### 2.1.2 Externalizing scale

The Externalizing scale consists of 12 items that assess under-controlled (e.g., verbal abuse, symptoms of impulsivity) and outer-directed manifestations of symptoms (Lau et al., 2021). The 5 under-controlled items are coded based on presence in the last 3 days (*0* = *not present; 4* = *exhibited daily in last 3 days, 3 or more episodes or continuously*). The 7 outer-directed items are scored according to a 6-point scaling system (*0* = *never; 5* = *in the last 3 days*). All scores were then dichotomized: 0 = item absent (score of 0); 1 = item present (scores > 1). A total score was computed by summing all 12 items; scores range from 0 to 12 with greater scores indicating higher levels of externalizing symptoms. The externalizing scale has demonstrated strong content (i.e., expert panel) and criterion validity with externalizing scores from other measures (e.g., Child Behavior Checklist, *r* = 0.65). Internal consistency for the externalizing scale was Cronbach's α = 0.86.

#### 2.1.3 Sleep disturbances

The Sleep Disturbances measure is comprised of 4-items: (a) difficulty falling asleep or staying asleep (e.g. waking up too early or restlessness), (b) waking up multiple times at night, (c) falling asleep during the day (not including nap time), and (d) resisting bedtime (e.g., not wanting to sleep at appropriate time or difficulty sleeping without caregiver intervention). Informants were asked about the frequency of specific sleep problems over the last 3 days (*0* = *not present; 4* = *exhibited daily in last 3 days, 3 or more episodes or continuously*). Items were summed to yield a total score ranging from 0 to 16 with higher scores indicating a greater degree of sleep difficulty. Clinically high sleep disturbance scores are > 9. The four sleep disturbance items are best conceptualized as an index, as the individual items contribute to sleep disturbances as a whole but difficulties are not assumed to co-occur (Bollen and Bauldry, 2011; Fayers and Hand, 1997; Hardin et al., 2010). As such, internal consistency for the sleep items was not computed.

The psychometric properties of this scale had not been examined. Thus, in a separate study we recruited 50 participants from child and adolescent mental health agencies that used the interRAI ChYMH. Parents were recruited for the study after they completed the interRAI ChYMH and then completed the Sleep Disturbance Scale for Children (SDSC, Bruni et al., 1996) via an on-line survey. Their data were matched with their interRAI ChYMH responses. The SDSC was positively correlated (*r* = 0.66) with parent-reported sleep disturbance on the interRAI ChYMH. Of note, in this small subsample 78% had scores above the clinical cut-off (i.e., 39) on the SDSC.

The Adolescent Supplement—In addition to the items noted above regarding sleep, an additional self-report item is also completed for adolescents (aged 12+ years); clinicians completing the measure may also include children under 12 years who are engaging in more mature behaviors (e.g., substance use, sexual activity). Adolescents are asked how they would rate their general sleep quality (*0* = *excellent, 3* = *poor*), with higher scores indicating poorer subjective sleep. Previous research has shown that parents tend to idealize adolescent sleep, and that adolescent self-report of sleep disturbances are more severe and more in line with objective measures of sleep disturbance, such as actigraphy (Short et al., 2013).

### 2.2 Covariates

#### 2.2.1 Individual-level covariates

Child birthyear and sex (male, female, other) were recorded at the time of the interRAI ChYMH assessment. Age was calculated based on birth year and year of the assessment.

Pain severity was measured by two items: (a) frequency of pain was assessed by how often the child/youth shows evidence of, or complains of, pain (*0* = *none, 3* = *exhibited daily in the last 3 days*); (b) intensity of pain was the highest level of pain present in the specified time period (*0* = *no pain, 4* = *times when pain is horrible or excruciating*). A total pain score was computed by averaging scores across the 2 items, higher scores indicate higher pain severity.

Sensory Sensitivity was measured by three items on excessive responses to stimuli (i.e., excessive or unusual reaction to sensory stimuli, sensory seeking behavior, sensory avoiding behavior). A total score was formed by averaging across items; scores range from 0 to 4 with higher scores indicating greater sensory sensitivity.

#### 2.2.2 Family-level covariates

Caregiver Distress (CD) consisted of 3-items related to stress and trauma experienced by parents or primary caregivers (hereafter caregivers). Items were scored as either present (1) or absent (0) and include: (a) major life stressors for caregivers in the last 90 days (e.g., death or severe illness of close family member/friend, loss of home); (b) caregiver unable or unwilling to continue in caring activities; and (c) caregiver expresses feelings of distress, anger, or depression. A CD score was the sum of the 3 items; scores range from 0 to 3 with higher scores indicating greater caregiver distress.

The Family Functioning Index consisted of 6-items that measure different aspects of family functioning based on the presence (1) or absence (0) of: (a) family that are persistently hostile or critical of child/youth; (b) family reporting feeling overwhelmed by child/youth's condition; (c) caregiver with current developmental or mental health issue; (d) sibling with current developmental or mental health issues; (e) caregiver unable or unwilling to continue in caring activities; and (f) a strong and supportive relationship with family. A total score was the sum of all items; scores range from 0 to 5 with higher scores indicating greater family dysfunction.

The Parenting Strengths scale consisted of 6-items rated on the frequency of interaction between caregiver and child/youth, taking into account developmental age of the child (*0* = *most of the time, 2* = *rarely or never*). Items included: (a) effective communication, (b) assistance with emotion regulation, (c) appropriate discipline, (d) warmth and support demonstrated, (e) supervision and monitoring as appropriate, and (f) appropriate expectation or limit setting. Items were reverse coded to assess the lack of parenting strengths and summed to compute a total score ranging from 0 to 12. Internal consistency was good: Cronbach's α = 0.89.

### 2.3 Data analysis

#### 2.3.1 Missing data

For primary measures, considered essential for the current study, cases were removed if they were missing one of sleep, internalizing, or externalizing (*N* = 246). Other variables were considered secondary. Cases were removed if they were missing parenting strengths. For other secondary measures, cases were excluded if two or more measures were missing from family functioning, caregiver distress, or pain. A measure was considered missing if more than 15% of the individual items were missing.

Overall, 222 cases were excluded due to missing secondary measures, either missing the parenting strengths measure or missing two of the family functioning, parenting strengths, or pain measures (see Figure 1). Of participants included in the final sample, 0.3% were missing the caregiver distress score (*n*= 43) and 0.1% were missing a pain score (*n* = 7). Multiple imputation analyses were completed for participants missing data. Initial versions of the interRAI ChYMH did not include questions about sensory sensitivity, thus multiple imputation analysis was conducted in SPSS to account for this non-random missing information (*N* = 859). Cases with and without sensory sensitivity were compared on other study variables to ensure there were no significant differences.

#### 2.3.2 Hierarchical regressions

A split-sample approach was used to determine whether results are reproducible. In analyses where significance is found in only one sample, results were not considered robust findings; as such, only findings that were statistically significant in both split-samples are reviewed. The sample was broken down into two groups based on a random selection of approximately 50% of cases, Sample 1 (S1; *n* = 6,773) and Sample 2 (S2; *n* = 6,699).

Two hierarchical regression analyses were conducted for both outcomes (i.e., internalizing, externalizing): (1) using a sample of the entire age range, (2) for adolescents only (as self-reported sleep scores were not available for children).

Variables were entered in 5 blocks for each regression model. (1) Demographic variables (sex, age) were entered. (2) Family covariates (parenting strengths, caregiver distress, family functioning) were entered. (3) Individual covariates (pain, sensory sensitivity) were entered. (4) Sleep disturbances were entered. (5) For all ages, the interaction term (Age X Sleep disturbance) was included. Sleep disturbance and age were mean centered prior to computing the interaction term (Kreft et al., 1995). For adolescents only, adolescent reports of subjective sleep quality were included to determine whether sleep quality had an additional relationship to symptoms of psychopathology, above and beyond reported sleep disturbances.

When a significant Age X Sleep disturbance interaction was found, the interactive relationship was further investigated using the Preacher and Hayes PROCESS Macro for SPSS (Hayes, 2018; Preacher and Hayes, 2008). The interaction was examined after controlling for individual and family covariates and mean centering was used for age and sleep disturbance. Graphs of the Sleep disturbance X Age effects were plotted at the 16th, 50th and 84th percentile and simple slopes were examined. Finally, a three-way interaction effect (Age X Sex X Sleep distrubance) was invesigated using the same process as above.

## 3 Results

### 3.1 Descriptive statistics

A total of 13,472 children were included in the analyses with an average age 12.1 years (*SD* = 3.56); 55% were male. For both S1 and S2, the means and standard deviations are shown in Table 1.

**Table 1:** Means and standard deviations for sample 1 and sample 2.

	**Sample 1—All**			**Sample 2—All**			**Sample 1—Adolescent**			**Sample 2—Adolescent**		
**Variables**	***N***	***M***	**SD**	***N***	***M***	**SD**	***N***	***M***	**SD**	***N***	***M***	**SD**
1. Internalizing	6,773	10.26	8.83	6,699	10.24	8.79	3,375	11.78	9.44	3,372	11.73	9.37
2. Externalizing	6,773	4.57	3.11	6,699	4.61	3.13	3,375	4.09	3.29	3,372	4.08	3.23
3. Sex^a^												
a. Male	3,833	56.6%		3,729	55.7%		1,713	46.4%		1,660	45.1%	
b. Female	2,927	43.2%		2,952	44.1%		1,965	53.3%		2,005	54.4%	
4. Age^b^	6,773	12.12	3.57	6,699	12.17	3.56	3,690	14.89	1.71	3,683	14.91	1.75
5. Pain^e^	6,768	0.22	0.61	6,697	0.23	0.63	3,690	0.28	0.69	3,683	0.30	0.71
6. Parenting strengths^c^	6,773	10.35	2.65	6,699	10.30	2.67	3,690	9.87	3.00	3,683	9.79	3.02
7. Caregiver distress^e^	6,752	0.68	0.81	6,677	0.69	0.82	3,690	0.71	0.84	3,683	0.72	0.84
8. Family functioning^d^	6,773	1.41	1.21	6,699	1.44	1.22	3,690	1.52	1.25	3,683	1.54	1.28
9. Sensory sensitivity^e^	6,326	0.42	0.82	6,287	0.41	0.85	3,690	0.27	0.67	3,683	0.28	0.69
10. Sleep disturbance	6,773	3.40	3.54	6,699	3.40	3.49	3,690	3.44	3.59	3,683	3.51	3.56
11. Adolescent—sleep quality^f^		–	–		–	–	3,375	1.69	0.91	3,372	1.69	0.90

Data were randomly assigned to sample 1 or sample 2.

Scores are out of the following: internalizing (0–48), externalizing (0–12), pain (0–4), parenting strengths (0–12), caregiver distress (0–3), family functioning (0–5), sensory sensitivity (0–4), sleep (0–16), adolescent—subjective sleep (0 to 3).

^a^Sex was coded as follows: 1 = male, 0 = female, 2 = other.

^b^Age was calculated as year of assessment minus year of birth.

^c^Higher scores reflect lower levels of parenting strengths.

^d^Higher scores reflect lower levels of family functioning.

^e^If original value was missing average scores across 5 imputations were used.

^f^Variable only included in adolescent supplement for children ages 12 and older.

The inter-correlations of variables are reported, separately for S1 and S2, in Table 2. For the majority of cases, correlations were significant in both S1 and S2.

**Table 2:** Correlations among sample 1 variables (below diagonal) and among sample 2 variables (above diagonal).

**Variables**	**1**	**2**	**3**	**4**	**5**	**6**	**7**	**8**	**9**	**10**	**11**
1. Internalizing		0.14^**^	0.15^**^	0.24^**^	0.19^**^	−0.10^**^	0.18^**^	0.23^**^	0.17^**^	0.40^**^	0.30^**^
2. Externalizing	0.14^*^		−0.26^**^	−0.20^**^	0.01	−0.19^**^	0.32^**^	0.37^**^	0.22^**^	0.21^**^	0.002
3. Sex^a^	0.17^**^	−0.21^**^		0.24^**^	0.09^**^	−0.06^**^	−0.03	0.00	−0.13^**^	0.05^**^	0.12^**^
4. Age^b^	0.24^**^	−0.18^**^	0.23^**^		0.14^**^	−0.22^**^	0.05^**^	0.09^**^	−0.24^**^	0.04^**^	0.16^**^
5. Pain	0.18^**^	0.02^*^	0.10^**^	0.12^**^		−0.07^**^	0.11^**^	0.09^**^	0.06^**^	0.16^**^	0.15^**^
6. Parenting Strengths^c^	−0.10^**^	−0.19^**^	−0.06^**^	−0.23^**^	−0.05^**^		−0.34^**^	−0.44^**^	0.09^**^	−0.12^**^	−0.11^**^
7. Caregiver Distress	0.19^**^	0.32^**^	0.00	0.05^**^	0.11^**^	−0.35^**^		0.50^**^	0.09^**^	0.21^**^	0.12^**^
8. Family Functioning^d^	0.24^**^	0.35^**^	0.06^**^	0.11^**^	0.09^**^	−0.46^**^	0.51^**^		0.07^*^	0.23^**^	0.14^**^
9. Sensory Sensitivity^e^	0.16^**^	0.21^**^	−0.12^**^	−0.23^**^	0.04^**^	0.07^**^	0.07^**^	0.07^**^		0.17^**^	0.05^**^
10. Sleep Disturbance	0.41^**^	0.23^**^	0.04^**^	0.02	0.15^**^	−0.11^**^	0.20^**^	0.24^**^	0.17^**^		0.49^**^
11. Adolescent – Sleep Quality^f^	0.30^**^	0.02	0.13^**^	0.17^**^	0.12^**^	−0.10^**^	0.10^**^	0.15^**^	0.00	0.51^**^	

^**^p ≤ 0.5, ^*^p ≤ 0.01.

Data were randomly assigned to Sample 1 (below diagonal) or Sample 2 (above diagonal). Due to tolerance of missing values in secondary measures and a subset of adolescent only data, N's vary across correlations. For Sample 1 data, N's are as follows: for all ages variables N = 6,773; for adolescent subjective sleep N = 3,375. For Sample 2 data, N's are as follows: for all ages variables N = 6,699; for adolescent subjective sleep N = 3,372.

^a^Sex was coded as follows: 1 = male, 0 = female, 2 = other.

^b^Age was calculated as year of assessment minus year of birth.

^c^Higher scores reflect lower levels of parenting strengths.

^d^Higher scores reflect lower levels of family functioning.

^e^If data was missing, reported value is the average across 5 imputations.

^f^This variable was only included in the adolescent supplement for children ages 12 and older.

InterRAI ChYMH sleep disturbance scores and adolescent self-reported sleep quality were correlated: *r* = 0.51 (S1); *r* = 0.49 (S2). For the total sample, on the sleep disturbances scale 33.4% of children and adolescents has moderately elevated scores (4–9/16) and 6.7% had high scores (i.e., > 9). For the internalizing scale, 39.5% of children and adolescents had clinically high scores (i.e., scores > 10). There are no clinical cut-off scores for the externalizing scale; 38.5% of the sample had externalizing scores at or above 6 (out of 12).

In the bi-variate analyses, higher levels of sleep disturbances were related with higher levels of both internalizing (*r* = 0.40-0.41, S1 and S2 respectively) and externalizing problems (*r* = 0.23-21; see Table 2). Interestingly, adolescent self-reported sleep quality was also correlated with internalizing (*r* = 0.30, S1 and S2), but not externalizing problems (*r* = 0.02-0.002).

### 3.2 Hierarchical regressions

Table 3 presents the final regression equations for internalizing symptoms and Table 4 shows results for externalizing symptoms. Sleep disturbances accounted for a significant proportion of the variance for internalizing symptoms (S1 and S2, ΔR^2^ = 0.10) and a small but statistically significant proportion of the variance for externalizing symptoms (S1 ΔR^2^ = 0.02, S2 ΔR^2^ = 0.01), after controlling for demographics, individual and family variables in accordance with our hypotheses.

**Table 3:** Hierarchical multiple regression: internalizing symptoms.

	**Sample 1–All**		**Sample 2–All**		**Sample 1—Adolescent**		**Sample 2—Adolescent**	
**Variable**	β		β		β		β	
***Step 1: demographics***								
Sex^a^	0.12^***^		0.10^***^		0.20^***^		0.16^***^	
Age^b^	0.21^***^		0.22^***^		0.11^***^		0.12^***^	
*ΔR^2^*		0.07		0.07		0.06		0.04
*ΔF*	252.52^***^		239.05^***^		101.79^***^		78.98^***^	
***Step 2: family variables***								
Parenting strengths^c^	0.08^***^		0.07^***^		0.06^**^		0.04^*^	
Caregiver distress	0.11^***^		0.10^***^		0.12^***^		0.06^**^	
Family functioning^d^	0.19^***^		0.20^***^		0.16^***^		0.19^***^	
*ΔR^2^*		0.06		0.06		0.05		0.04
*ΔF*	143.70^***^		142.73^***^		63.51^***^		55.62^***^	
***Step 3: individual variables***								
Pain	0.11^***^		0.12^***^		0.12^***^		0.13^***^	
Sensory sensitivity^e^	0.20^***^		0.21^***^		0.14^***^		0.18^***^	
*ΔR^2^*		0.05		0.06		0.04		0.05
*ΔF*	209.70^***^		230.03^***^		73.18^***^		103.32^***^	
***Step 4: sleep disturbances***								
Sleep disturbance	0.33^***^		0.32^***^		0.35^***^		0.34^***^	
*ΔR^2^*		0.10		0.10		0.11		0.10
*ΔF*	926.44^***^		827.23^***^		494.89^***^		467.59^***^	
***Step 5: age as a moderator***								
Sleep x age	0.07^***^		0.07^***^		-		-	
*ΔR^2^*		0.004		0.004		-		-
*ΔF*	41.84^***^		39.76			-		-
***Step 5: sleep quality***								
Adolescent sleep quality^f^	-		-		0.10^***^		0.10^***^	
*ΔR^2^*		-		-		0.01		0.01
*ΔF*		-		-	29.74^***^		29.88^***^	

^*^p ≤ 0.5, ^**^p ≤ 0.01, ^***^p ≤ 0.001.

β values reported for model 5 only.

^a^Sex was coded as follows: 1 = male, 0 = female, 2 = other.

^b^Age was calculated as year of assessment minus year of birth.

^c^Higher scores reflect lower levels of parenting strengths.

^d^Higher scores reflect lower levels of family functioning.

^e^Analyses were conducted using values from 5 imputations and the average value reported.

^f^Variable only included in adolescent supplement for children ages 12 and older.

**Table 4:** Hierarchical multiple regression: externalizing symptoms.

	**Sample 1—All**		**Sample 2—All**		**Sample 1—Adolescent**		**Sample 2—Adolescent**	
**Variable**	β		β		β		β	
***Step 1: demographics***								
Sex^a^	−0.18^***^		−0.23^***^		−0.19^***^		−0.26^***^	
Age^b^	−0.14^***^		−0.14^***^		−0.11^***^		−0.10^***^	
*ΔR^2^*		0.06		0.09		0.05		0.08
*ΔF*	224.88^***^		323.73^***^		94.63^***^		155.82^***^	
***Step 2: family variables***								
Parenting strengths ^c^	−0.06^***^		−0.06^***^		−0.08^***^		−0.09^***^	
Caregiver distress	0.17^***^		0.16^***^		0.18^***^		0.16^***^	
Family functioning^d^	0.26^***^		0.28^***^		0.25^***^		0.26^***^	
*ΔR^2^*		0.17		0.17		0.18		0.18
*ΔF*	494.81^***^		525.91^***^		258.43^***^		266.00^***^	
***Step 3: individual variables***								
Pain	−0.01		−0.004		0.02		0.01	
Sensory sensitivity^e^	0.12^***^		0.13^***^		0.10^***^		0.11^***^	
*ΔR^2^*		0.02		0.02		0.01		0.01
*ΔF*	66.80^***^		67.96^***^		24.25^***^		28.65^***^	
***Step 4: sleep disturbances***								
Sleep disturbance	0.14^***^		0.12^***^		0.12^***^		0.09^***^	
*ΔR^2^*		0.02		0.01		0.01		0.01
*ΔF*	152.23^***^		114.17^***^		57.08^***^		37.11^***^	
***Step 5: Age as a moderator***								
Sleep x age	−0.01		−0.01		-		-	
*ΔR^2^*		0.00		0.00		-		-
*ΔF*	0.29		1.70^***^			-		-
***Step 5: sleep quality***								
Adolescent sleep quality^f^	-		-		−0.08^***^		−0.08^***^	
*ΔR^2^*		-		-		0.004		0.01
*ΔF*		-		-		18.25^***^		23.16^***^

^*^p ≤ 0.5, ^**^p ≤ 0.01, ^***^p ≤ 0.001.

β values reported for model 5 only.

^a^Sex was coded as follows: 1 = male, 0 = female, 2 = other.

^b^Age was calculated as year of assessment minus year of birth.

^c^Higher scores reflect lower levels of parenting strengths.

^d^Higher scores reflect lower levels of family functioning.

^e^Analyses were conducted using values from 5 imputations and the average value reported.

^f^Variable only included in adolescent supplement for children ages 12 and older.

Among adolescents, after controlling for demographics, individual and family variables, and sleep disturbances, self-reported subjective sleep quality explained a small but significant amount of the variance in internalizing symptoms (S1 and S2, ΔR^2^ = 0.01).

For externalizing symptoms, subjective sleep quality was statistically significant; however, the direction of the relationship in the regression (Table 4) is opposite to the bivariate correlation (Table 2). This indicates a suppressor effect and, as such, it is not appropriate to interpret the relationship (Maasen and Bakker, 2001; Tzelgov and Henik, 1991).

### 3.3 Age as a moderator

The Age X Sleep disturbances interaction added a small (S1 and S2, ΔR^2^ = 0.004), but statistically significant proportion of the variance for internalizing symptoms after controlling for demographics, family variables and individual variables in accordance with our hypothesis. However unlike hypothesized, for externalizing, the interaction term was non-signifiant in both S1 and S2.

**Figure 2:**
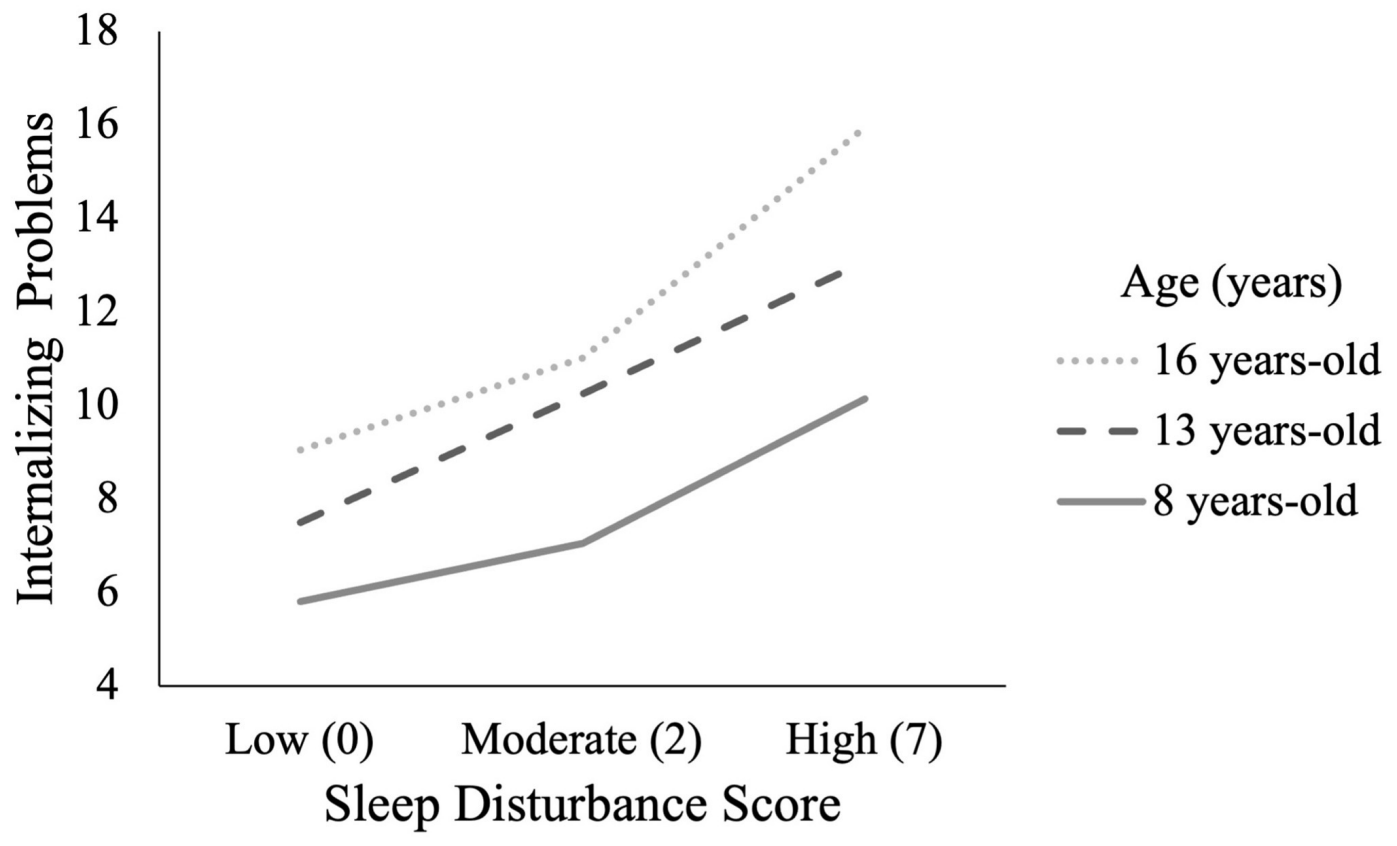
Relationship between internalizing and sleep by age group. Age was plotted in years at the 16th percentile (8.0; low), 50th percentile (13.0; moderate) and 84th percentile (16.0; high). Sleep sum score was also plotted at the 16th percentile (0.0; low), 50th percentile (2.0; moderate) and 84th percentile (7.0; high).

We investigated the interactive effects of age and sleep disturbance on internalizing symptoms using PROCESS Macro in the full sample. Consistent with regressions reported above, the overall model, including all covariates, was significant [F_(9, 13462)_= 572.24*, p* < 0.001], with an *R*^2^ of 0.28. The addition of the Age X Sleep disturbance interaction term was also significant (β = 0.05, *p* < 0.001) with a Δ*R*^2^ of 0.004. We then plotted the simple slopes of this relationship at ages 8, 13 and 16 years, which reflects the 16th, 50th and 82nd percentiles of age (see Figure 2). Standardized coefficients were significant (*p*'s > 0.001) at each age, with the magnitude of the effect increasing with older age: β = 0.61 (8 years), β = 0.85 (13 years), and β = 0.99 (16 years). This finding was aligned with our hypothesis. This indicates that the relationship between sleep disturbance and internalizing becomes more pronounced in later adolescence vs. childhood. Contrary to our hypotheses, the three-way interaction between age, sex and sleep disturbance on internalizing symptoms was non-significant [F_(1, 13385)_ = 0.0855*, p* = 0.77, ΔR^2^ = 0.00].

## 4 Discussion

Amongst children and adolescents being assessed as part of routine clinical services in children's mental health agencies from across Ontario, higher levels of sleep disturbances were related to both internalizing and externalizing symptomology. These relationships were significant after controlling for other individual–(age, sex, pain, sensory sensitivity) and family-level (i.e., parenting strengths, caregiver distress, family functioning) factors that impact both sleep issues and psychopathology. The strength of this relationship was greater for internalizing than externalizing problems, and the relationship between sleep disturbances and internalizing becomes stronger as children move through adolescence. Previous studies have not controlled for multiple individual- or family-variables that could contribute to both sleep and psychopathology. Thus, our findings strengthen the importance of considering the impact of sleep disturbances among children and adolescents with mental health problems and highlight the need to focus on the importance of sleep and internalizing disorders among adolescents.

### 4.1 Sleep disturbances and psychopathology

The results of the current study showed higher sleep disturbance scores were related to higher internalizing scores, which is consistent with past population-based studies. Two recent reviews found that sleep problems during childhood were related to both anxiety and depression during adolescence (Lam and Lam, 2021; Marino et al., 2021). Our findings are also consistent with samples of children with mental health problems. Some studies have focused on the prevalence and/or the impact of sleep problems in samples of children with anxiety or depression (e.g., Alfano et al., 2007), while other studies report that sleep problems are amongst the most common symptoms, particularly amongst adolescents (e.g., Goodyer et al., 2017; Orchard et al., 2017). Higher levels of sleep problems (e.g., troubles with sleep onset, long duration night waking) before receiving mental health treatment are related to higher levels of internalizing problems (Bai et al., 2020; Caporino et al., 2017), and both shorter time to and greater likelihood of recurrence of depression (Emslie et al., 2001). Cross-lagged analyses of the relationship between internalizing and sleep problems after completion of mental health treatment found that more sleep problems (e.g., trouble sleeping, sleeplessness) predicted more severe internalizing problems, and vice versa; however, neither changes in sleep problems nor changes in internalizing problems were related to each other (Bai et al., 2020). Our findings and those of past studies suggest that greater sleep problems at the start of mental health treatment may be contributing to more severe internalizing problems.

### 4.2 Age as a moderator

Consistent with our hypothesis, age moderated the relationship between sleep distrubance and internalizing, although the overall percentage of variance explained by the interaction was not large. Longitudinal research looking at the reciprocal relationship of sleep difficulties and anxiety and depression has found similar results (Gregory and O'Connor, 2002; Alfano and Gamble, 2009; Beebe, 2011; Van Dyk et al., 2016; Vazsonyi et al., 2022). This change in reciprocal relationships could in part be due to interactions changing across developmental periods or difficulties during critical periods in development (Kelly and El-Sheikh, 2014). Although specific mechanisms have not been well established, the role of biological, psychological, and social mechanisms underlying the relationship between sleep disturbances and internalizing disorders in adolescence has been suggested.

For children, separation-related sleep difficulties are more strongly related to anxiety among children, compared to adolescence (Caporino et al., 2017), and separation anxiety is more common among young children (Copeland et al., 2014). As children develop, they may be more able to cope with separation from parents, leading to reductions in both sleep- and anxiety-related separation issues. A review of mechanisms underlying the relationship between insomnia, and anxiety and depression, in adolescence found that among biological mechanisms, puberty and sleep deprivation may have an additive effect on reward processing, specifically increasing vulnerability to internalizing disorders among those experiencing sleep difficulties during adolescence (Blake et al., 2018). Psychological research has shown that among adolescents (age 11–19) with a sleep disorder, 87% reported catastrophic thinking prior to sleep, largely focused around interpersonal relationships at school and school performance (Hiller et al., 2014). As social pressures increase for adolescents entering high school, increased catastrophic thinking might contribute to both sleep and internalizing difficulties in this age group. Poor sleep might undermine behavioral and emotional regulation in adolescents and reduce their resources for coping in social and family situations. These limited coping mechanisms might be overwhelmed in adolescents experiencing high family stress and low socioeconomic status, thus disrupting interpersonal relationships and increasing risk for anxiety and depression (Blake et al., 2018). Biological and neurological changes interacting with social changes during adolescence have also been suggested as mechanisms through which sleep problems contribute to internalizing problems (Akbar et al., 2022).

Sleep disturbances also had a significant relationship to externalizing problems. Numerous studies (e.g., Steinsbekk and Wichstrom, 2015) including a recent systematic review (Liu et al., 2022) have found consistent evidence for a relationship between various aspects of sleep (e.g., sleep duration, sleep quality, insomnia) and externalizing problem, amongst children and adolescents, and in both cross-sectional and longitudinal studies. This relationship was stronger for internalizing than externalizing. Some studies that have examined sleep issues and both internalizing and externalizing in the same sample have also found the magnitude of the effects appear greater for internalizing (Yue et al., 2022). However, other studies have found both cross-sectional and longitudinal relationships are comparable in magnitude (Quach et al., 2018; Steinsbekk and Wichstrom, 2015; Shanahan et al., 2014). It should be noted that neither our study nor the studies cited examined if the parameters for internalizing vs. externalizing and sleep problems were statistically significantly different. It is possible that the relationship between sleep and specific externalizing problems, like ADHD and ODD, is driven by the shared feature of significant irritability (Shanahan et al., 2014).

### 4.3 Adolescent self-report sleep

Sleep problems may be underestimated by parent-report (e.g., Paavonen et al., 2002; Aronen et al., 2000). One study among adolescents (13–17) found differences in the percentage that met criteria for a sleep problem based on self- (21.1%) vs. parent-reports (14.3%; Short et al., 2013). We found that adolescent-reported sleep quality was significantly related to higher internalizing symptoms, above and beyond parent-rated sleep problems and other covariates. Sleep and internalizing problems have bi-directional relationship (see Bagley and El-Sheikh, 2013; Crowe and Spiro-Levitt, 2024). Our findings may reflect a process in which adolescents' psychological distress impacts their sleep quality, or vice versa. Their distress and related tendencies toward negative cognition in general could also lead them to perceive their sleep quality more poorly. Similar to community-based studies (Quach et al., 2018), cross-lagged correlational analyses of longitudinal data could help inform the direction of these relationships.

### 4.4 Implications

The current study may have implications related to addressing sleep problems in samples of children and adolescents with mental health problems. A qualitative study in the United Kingdom of Child and Youth Mental Health Services identified that sleep problems are often a problem, but rarely treated (Higson-Sweeney et al., 2020). There have been two recent reviews of the literature on sleep interventions for children and adolescents with mental health problems (Bourchtein et al., 2020; Dewald-Kaufmann et al., 2022). Although there are few studies, they concluded that nonpharmacological interventions have significant positive impacts on sleep issues for children and adolescents with internalizing or externalizing problems. Interventions may be particularly important for adolescents with higher levels of internalizing problems. In terms of secondary outcomes, sleep interventions have positive impacts on parent-rated behavior problems. Results for internalizing issues were mixed. A recent RCT targeting anxiety amongst adolescents found no change in sleep problems despite overall improvements in anxiety (Haugland et al., 2021). Another recent trial with children did not find any added benefit to addressing both sleep and anxiety on either anxiety or sleep outcomes (Clementi and Alfano, 2020). It is also important to note that having sleep problems at the end of treatment has been associated with increased risk for relapse amongst adolescents treated for depression (Manglick et al., 2013). In light of the current state of the literature our findings suggest that, for adolescents with high levels of internalizing problems, if sleep problems still exist at the end of treatment for their mental health problems, CBTi (Dewald-Kaufmann et al., 2022) should be considered to address residual sleep issues.

### 4.5 Limitations

Strengths of the current study include a large sample which allowed for split-half analyses that showed consistent findings. Use of administrative data avoids problems with possible sample biases when subjects are recruited specifically for research studies. There are, however, limitations. First, our measure of sleep disturbances is not one that is commonly used in other studies. A sub-study demonstrated reasonable correlation with an established parent-report measure of sleep problems. Within the small substudy sample, the vast majority of cases (~ 80%) were above clinical cutoffs on the SDSC; compared to the prevalence of clinically “high” sleep problems on the ChYMH (6.7%). The substudy provides additional validity support for the ChYMH sleep measure but raises questions about the clinical cut offs for the sleep disturbances scale. As is common in studies of clinical mental health samples, established sleep measures are not routinely administered, given that sleep is rarely the primary presenting problem in child and adolescent mental health clinics. Thus, from a clinical perspective, it is likely not helpful if the ChYMH were to flag 4 out of every 5 cases as having sleep issues. Future research is needed to better understand when sleep problems should be addressed for children and adolescents presenting to mental health clinics. Second, the ChYMH does not have norms from a population-based community sample. As such, we could not compare the prevalence of clinically-significant problems in our data with the general population. Third, our cross-sectional analyses could not examine the relationship between changes in sleep and changes in psychopathology, and vice versa. As noted above, future research using longitudinal designs is needed. Fourth, despite the benefits of using a clinical sample, other diagnoses could have impacted our measures, such as chronic medical conditions or physical sleep disorders such as obstructive sleep apnea. Fifth, in a mental health clinical sample we would expect that some individuals were prescribed medications that could have impacted their sleep; however, these data were not available. Finally, there are myriad factors that influence both sleep and psychopathology (Bartel et al., 2015; Newton et al., 2020). We controlled for all relevant covariates that were available in the dataset. However, there are certainly other important variables that we were not able to control for.

## Data Availability

The data analyzed in this study is subject to the following licenses/restrictions: The data is highly sensitive and confidential. Due to the ethical requirements required for use of the data at the present institution (i.e., data collected on secure server, VPN protected, password protected data in secure room with no access to internet or USB ports, etc.), data will not be made freely available. Additionally, the participating mental health agencies required that data not be made freely accessible, to protect the anonymity of participants. Requests to access these datasets should be directed to https://uwaterloo.ca/interrai-canada/.
